# Evaluation of Freehand B-Mode and Power-Mode 3D Ultrasound for Visualisation and Grading of Internal Carotid Artery Stenosis

**DOI:** 10.1371/journal.pone.0167500

**Published:** 2017-01-03

**Authors:** Johann Otto Pelz, Anna Weinreich, Thomas Karlas, Dorothee Saur

**Affiliations:** 1 Department of Neurology, Leipzig University Hospital, Leipzig, Germany; 2 Department of Gastroenterology and Rheumatology, Leipzig University Hospital, Leipzig, Germany; Heart Research Institute, AUSTRALIA

## Abstract

**Background:**

Currently, colour-coded duplex sonography (2D-CDS) is clinical standard for detection and grading of internal carotid artery stenosis (ICAS). However, unlike angiographic imaging modalities, 2D-CDS assesses ICAS by its hemodynamic effects rather than luminal changes. Aim of this study was to evaluate freehand 3D ultrasound (3DUS) for direct visualisation and quantification of ICAS.

**Methods:**

Thirty-seven patients with 43 ICAS were examined with 2D-CDS as reference standard and with freehand B-mode respectively power-mode 3DUS. Stenotic value of 3D reconstructed ICAS was calculated as distal diameter respectively distal cross-sectional area (CSA) reduction percentage and compared with 2D-CDS.

**Results:**

There was a trend but no significant difference in successful 3D reconstruction of ICAS between B-mode and power mode (examiner 1 {Ex1} 81% versus 93%, examiner 2 {Ex2} 84% versus 88%). Inter-rater agreement was best for power-mode 3DUS and assessment of stenotic value as distal CSA reduction percentage (intraclass correlation coefficient {ICC} 0.90) followed by power-mode 3DUS and distal diameter reduction percentage (ICC 0.81). Inter-rater agreement was poor for B-mode 3DUS (ICC, distal CSA reduction 0.36, distal diameter reduction 0.51). Intra-rater agreement for power-mode 3DUS was good for both measuring methods (ICC, distal CSA reduction 0.88 {Ex1} and 0.78 {Ex2}; ICC, distal diameter reduction 0.83 {Ex1} and 0.76 {Ex2}). In comparison to 2D-CDS inter-method agreement was good and clearly better for power-mode 3DUS (ICC, distal diameter reduction percentage: Ex1 0.85, Ex2 0.78; distal CSA reduction percentage: Ex1 0.63, Ex2 0.57) than for B-mode 3DUS (ICC, distal diameter reduction percentage: Ex1 0.40, Ex2 0.52; distal CSA reduction percentage: Ex1 0.15, Ex2 0.51).

**Conclusions:**

Non-invasive power-mode 3DUS is superior to B-mode 3DUS for imaging and quantification of ICAS. Thereby, further studies are warranted which should now compare power-mode 3DUS with the angiographic gold standard imaging modalities for quantification of ICAS, i.e. with CTA or CE-MRA.

## Introduction

Worldwide, ischemic stroke is among the leading causes for disability, mortality and of great socio-economic importance [1, 2]. Large-artery atherosclerosis, i.e. an occlusion or stenosis with ≥ 50% diameter reduction of a brain-supplying artery, was shown to be the most common cause of stroke in middle-aged patients in central Europe (Germany) and has the highest rate of early stroke recurrence [3]. Therefore, screening for stenosis of brain-supplying arteries is mandatory in acute stroke patients [4, 5], with special emphasis on the origin of internal carotid artery (ICA) a preferential site for severe atherosclerosis. Digital subtraction angiography (DSA) is still considered as the “gold standard” imaging modality for grading ICA stenosis (ICAS) and for determining a patient’s eligibility for carotid endarterectomy (CEA) or carotid angioplasty and stenting (CAS) [4]. But given its invasive character with a frequency of periprocedural neurological complications of up to 2.6% [6], DSA is just recommended if non-invasive imaging modalities like two-dimensional colour-coded duplexsonography (2D-CDS), computed tomography angiography (CTA) or contrast-enhanced magnetic resonance angiography (CE-MRA) have yielded discordant results in before [4]. Three-dimensional ultrasound (3DUS) which is nowadays already routinely used in obstetrics [7] has the potential to visualise extracranial brain-supplying arteries similar to CTA or MRA and might ideally complement 2D-CDS which grades ICAS by predominantly assessing hemodynamic parameters [8, 9]. Studies that evaluated 3DUS for quantification of ICAS showed promising results (in chronological order [10–18]). However, there are still unsolved questions that might hamper translation of vascular 3DUS from bench to clinical routine. First, it is unclear which ultrasound mode should be the basis for 3DUS, e.g. 3DUS based on high-resolution native B-mode ultrasound [11, 16, 18] or 3DUS based on power mode [10, 12, 14, 15, 17]. Together with recent advances in (3D) ultrasound technology, native B-mode ultrasound allows visualisation of carotid vessels with high temporal and spatial resolution [18], but hypoechogenic or heavily calcified plaques still remain important limitations and restricted 3D visualisation of ICAS in 16% of cases in our previous study [18]. On the other hand, spatial resolution of power mode ultrasound is considerably lower than of high-resolution B-mode ultrasound [19] which might affect accuracy of measurements especially within stenotic lesions. Secondly, a great advantage of 3DUS compared to DSA is that 3DUS allows assessment of ICAS as both diameter and cross-sectional area (CSA) reduction with the latter being independent from the chosen projection view. Thus, assessment of CSA reduction might result in a better accuracy and inter-rater agreement of stenotic value. So far, studies which addressed assessment of stenotic value of ICAS did not compare both measuring methods systematically [10–18].

Therefore, the aim of this study was to evaluate 3DUS for quantification of ICAS in comparison to 2D-CDS. We expected that successful 3D reconstruction of ICAS would be higher when based on power mode ultrasound than on high-resolution B-mode ultrasound. Furthermore, due to the optimized visualisation of the vessel’s lumen, power-mode 3DUS might result in better inter-rater, inter-rater and, when compared to 2D-CDS, inter-method agreement than high-resolution B-mode ultrasound. Since stenotic value of ICAS was assessed as distal diameter and as distal CSA reduction percentage, we can analyse our data with both methods.

## Methods

The study was approved by the local Ethics Committee of the Medical Faculty of the University of Leipzig (reference number: 120-13-22042013) and all participants gave their written informed consent.

### Study population

From June 2013 to July 2014, 37 patients with 43 ICAS (23 male; median age 73 years, range 53–91 years) were consecutively recruited from our outpatient clinic (28 patients) and stroke unit (9 patients). Six of 37 patients had bilateral ICAS of whom 4 had low-degree contralateral ICAS and only 2 had hemodynamically relevant ICAS on both sides. All patients from our outpatient clinic were in regular control of a known ICAS and free of focal neurological symptoms ipsilateral to the ICAS over at least the previous 6 months, i.e. the ICAS was considered to be asymptomatic. The remaining 9 patients were treated on our stroke unit due to an acute ischaemic stroke (8 patients) or a transient ischaemic attack (1 patient) in the territory of the ICAS, i.e. they had a symptomatic ICAS. In the latter group of patients with a symptomatic ICAS, ICAS was initially diagnosed by CTA in 7 patients and by CE-MRA in 2 patients and confirmed by 2D-CDS before inclusion into the study. None of them had previously had CEA or CAS of the stenotic ICA.

### Three-dimensional ultrasound scanning

**Fig 1:**
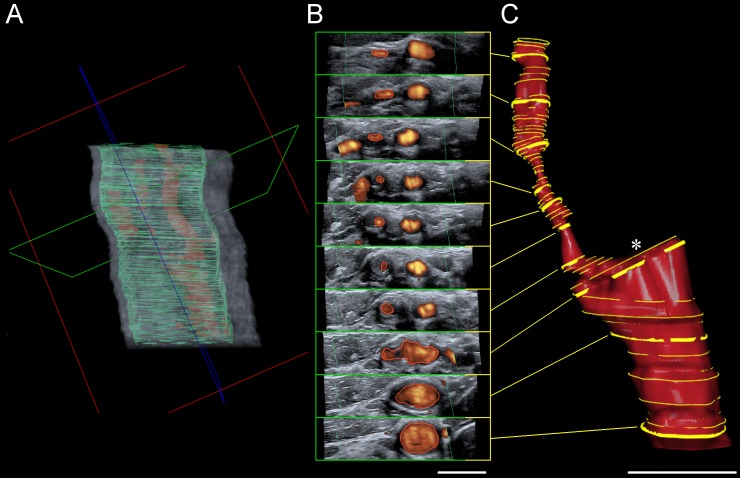
Three-dimensional ultrasound (3DUS) vessel reconstruction. (**A**) Two-dimensional power mode images were grabbed from the video port of the ultrasound system and concatenated with spatial and temporal information to be stored in a virtual 3D-volume. Within this virtual 3D-volume navigation in all 3 orthogonal spatial planes is possible ad libitum. Plane orientation: green = transversal, red = coronal, blue = sagittal. (**B**) Based on the transversal planes (green), the lumen of the common and internal carotid artery was traced manually at variable distances of 1 to 4 mm with smaller (1 mm) intersection intervals at level of the stenosis. This manual segmentation (all yellow lines) was followed by an automatic vessel reconstruction (**C**). The reconstructed carotid artery can be rotated freely and cross-sectional area can be measured at every point perpendicular to the vessel’s course. Asterisk indicates origin of external carotid artery (not shown). Scale bar each 1 cm.

All B-mode respectively power-mode 3DUS scans were performed by an experienced vascular neurologist (JP) using a Toshiba Aplio 500 (Toshiba Medical Systems GmbH, Neuss, Germany) equipped with a linear transducer (PLT-1204BT) set at 13 MHz This conventional ultrasound system was attached to the Curefab CS system (Curefab Technologies GmbH, Munich, Germany) which comprised a freehand magnetic field tracking system and a workstation equipped with special software (Curefab CS, version 1.91). This way, common 2D B-mode as well as power mode images, which were grabbed from the video port of the Toshiba Aplio 500, were concatenated with spatial and temporal information and stored in a virtual 3D-volume. Within this virtual 3D-volume, carotid arteries were reconstructed manually as visualized in Fig 1. The whole process of ICA visualisation took about 5 minutes in case of power-mode 3DUS and 10 minutes in case of B-mode 3DUS.

Practically, all patients were lying in a supine position and were asked not to swallow during the scan, while breathing was allowed. Various US parameters like gain and dynamic range (B-mode) respectively colour gain and latency (power-mode) as well as focus were individually optimised for each patient. In case of power-mode the setting was considered optimal when the patent lumen of the non-stenotic distal common carotid artery was completely filled with colour without relevant blooming artefacts or random noise in the adjacent tissue [19]. Subsequently, the ultrasound transducer was set cranial to the clavicle and medial to the sternocleidomastoid muscle and was moved cephalad in transversal direction keeping the ICA lumen in the center of the monitor screen. The whole scan took about 7 to 10 seconds and 36 (B-mode) or 13 (power mode) images per second were recorded. Altogether, each ICA was scanned six times, i.e. three B-mode 3DUS scans followed by three power-mode 3DUS scans. The best scan of each mode was used for post-processing. In addition, ICAS were also reconstructed from the second-best power-mode 3DUS scan to obtain intra-rater agreement for each examiner.

### Grading of ICAS by 2D-CDS und 3DUS

After anonymization of the data, patients were examined once by the same experienced vascular neurologist (JP) who was certified in neurosonology by the “German Society of Ultrasound in Medicine and Biology” (DEGUM) and the “European Society of Neurosonology and Cerebral Hemodynamics” (ESNCH). ICAS were graded with 2D-CDS applying the multiparametric “DEGUM ultrasound criteria” [8] which are almost identical to the criteria of the Neurosonology Research Group of the World Federation of Neurology [9].

**Fig 2:**
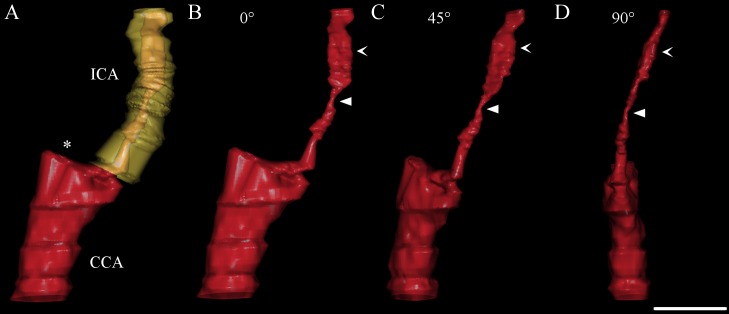
Stenotic value of internal carotid artery stenosis (ICAS) depends on projection view. (**A** and **B**) Anterior-posterior projection of power-mode 3D reconstructed ICAS; original lumen is also reconstructed and visualised in yellow (**A**). (**B—D**) Stenotic value of ICAS assessed as distal cross-sectional area reduction percentage (93%) does not change since it is not affected by rotation or tilting. By contrast, clockwise rotation of ICAS results in a decrease of stenotic value assessed as distal diameter reduction percentage from 82% (**B**) respectively 83% (**C**) to 72% (**D**). Smallest luminal diameter is marked with filled arrowhead while distal luminal diameter is marked with open arrowhead. Asterisk indicates origin of external carotid artery (not shown). CCA common carotid artery. Scale bar: 1 cm.

All subsequent 3DUS analyses (JP and AW) were done blinded to the results of the 2D-CDS examination as well as to the 3DUS results of the other examiner. For 3DUS, smallest luminal CSA within the stenosis as well as poststenotic normal luminal CSA were assessed perpendicular to the remaining lumen. Poststenotic lumen was considered as normal when there was again a complete colour filling of the lumen in the 3D-volume. Stenotic value of ICAS was then calculated as distal *CSA* reduction percentage. In addition, reconstructed ICA was rotated to obtain smallest luminal diameter within the stenosis and a screenshot was taken (Fig 2). Thereby, we aimed at simulating DSA with its 2D projection of a 3D structure. Luminal diameter was measured within ICAS and distal to it with ImageJ (1.48v; National Institutes of Health, Bethesda, MD, USA) and stenotic value was calculated as distal *diameter* reduction percentage according to the North American Symptomatic Carotid Endarterectomy Trial (NASCET) [20].

### Statistical analysis

Statistical analyses were performed with SPSS version 20.0 (IBM Corporation; New York, NY, USA). Intraclass correlation coefficient (ICC, consistency mode) was calculated to assess intra-rater and inter-rater agreement for stenotic value using B-mode respectively power-mode 3DUS. Visualisation and description of inter-method agreement between 2D-CDS and B-mode respectively power-mode 3DUS for grading of ICAS was achieved by a Bland and Altman analysis [21] and calculation of ICC since we assumed that in case of grading ICAS with 3DUS, which is an angiographic imaging modality free of hemodynamical constraints from the contralateral side, independence of both sides was given. Intraclass correlation coefficient (range of 0–1) was interpreted as follows: good agreement ICC ≥ 0.75, moderate agreement 0.75 < ICC ≥ 0.50 and poor agreement ICC < 0.5 [22]. Generally, a p < 0.05 was considered as statistical significant.

## Results

Thirty-seven patients with 43 ICAS were examined with 2D-CDS and 3DUS. Applying 2D-CDS as reference standard 15 of 43 (35%) ICAS were graded as ≥ 70% or high-grade. Six patients had bilateral ICAS, and 2 of those 6 patients had high-grade ICAS on both sides.

**Fig 3:**
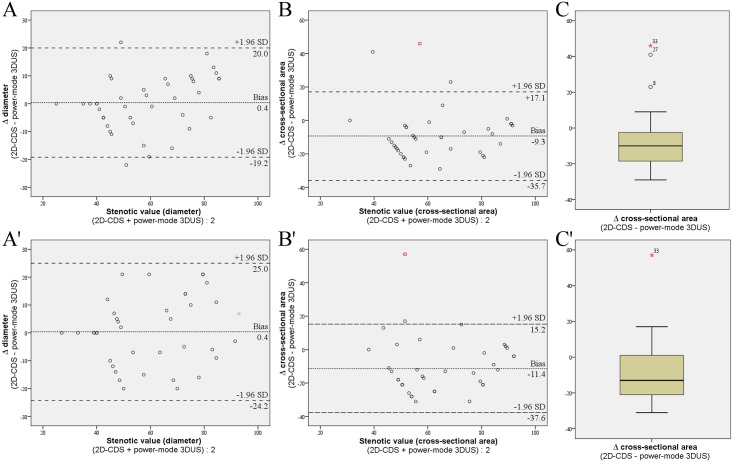
Inter-method agreement between power-mode 3DUS and 2D-CDS. **(A** and **B**) Inter-method agreements between power-mode 3DUS and 2D-CDS for grading ICAS are visualised by Bland and Altman analyses with the differences in stenotic values—assessed as distal *diameter* (**A** = examiner 1 and **A’** = examiner 2) respectively *CSA* (**B** = examiner 1 and **B’** examiner 2) reduction percentage—plotted against the mean stenotic value of both modalities. For the comparison of stenotic values assessed as distal *diameter* reduction percentage with 2D-CDS the Bland and Altman analyses (**A** and **A’**) showed no evidence of bias between methods but moderate limits of agreement (**A** bias 0.4%, limits of agreement 20.0 and -19.2; **A’** bias 0.4, limits of agreement 25.0 and -24.2). Assessment of stenotic value with power-mode 3DUS as distal *CSA* reduction percentage accounted for a permanent overestimation of ICAS in comparison to 2D-CDS and wide limits of agreement (**B** bias -9.3, limits of agreement 17.1 and -35.7; **B’** bias -11.4, limits of agreement 15.2 and -37.6). Note that ICAS number 33 (marked in red) was assumed to be an outlier when assessing stenotic value via CSA reduction percentage by both examiners as shown in the box-and-whisker plots (**C** and **C’**). Hence, Bland and Altman analyses with stenotic value assessed as distal CSA reduction percentage (**B** and **B’**) were performed without ICAS number 33. 3DUS three-dimensional ultrasound, 2D-CDS 2D colour-coded duplexsonography, ICAS internal carotid artery stenosis, SD standard deviation.

Three-dimensional vessel reconstruction from the virtual B-mode 3D-volume was possible in 35 of 43 ICAS (81%; examiner 1 {Ex1}) and 36 of 43 ICAS (84%; Ex2). Using power-mode 3DUS successful vessel reconstruction increased to 40 of 43 ICAS (93%, Ex1) and 38 of 43 ICAS (88%, Ex2) which was statistically not significant (chi-square test p = 0.11 {Ex1} and 0.39 {Ex2}). Reasons for failure of ICAS 3D-reconstruction were heavy calcification with acoustic shadowing and in case of B-mode 3DUS also echolucent plaques which could not be discriminated from the lumen. Intra-rater, inter-rater and inter-method agreement for high-resolution B-mode respectively power-mode 3DUS and 2D-CDS are shown in Table 1. For power-mode 3DUS, comparison of stenotic values assessed as distal *diameter* reduction percentage with 2D-CDS showed no evidence of bias between methods but moderate limits of agreement in the Bland and Altman analyses (Fig 3A). However, assessment of stenotic value with power-mode 3DUS as distal *CSA* reduction percentage accounted for a permanent overestimation of ICAS in comparison to 2D-CDS and wide limits of agreement (Fig 3B).

**Table 1:** Intra-rater, inter-rater and inter-method agreement of 3D ultrasound (3DUS) for quantification of internal carotid artery stenosis (ICAS).

	Intra-rater agreement (ICC)		Inter-rater agreement (ICC)		Inter-method agreement with 2D-CDS for Ex1(ICC)		Inter-method agreement with 2D-CDS for Ex2 (ICC)	
	Ex1 (1. & 2. scan)	Ex2 (1. & 2. scan)	1. scan	2. scan	1. scan	2. scan	1. scan	2. scan
B-mode 3DUS: distal CSA reduction percentage	n.a.	n.a.	0.36* (CI: 0.27–0.44)	n.a.	0.15 (CI: 0.09–0.20)	n.a.	0.51* (CI: 0.44–0.60)	n.a.
B-mode 3DUS: distal diameter reduction percentage	n.a.	n.a.	0.51* (CI: 0.43–0.58)	n.a.	0.40* (CI: 0.31–0.47)	n.a.	0.52* (CI: 0.46–0.59)	n.a.
power-mode 3DUS: distal CSA reduction percentage	0.88* (CI: 0.79–0.94)	0.78* (CI: 0.62–0.88)	0.90* (CI: 0.82–0.95)	0.8* (CI: 0.70–0.91)	0.63* (CI: 0.40–0.79)	0.63* (CI: 0.39–0.78)	0.57* (CI: 0.33–0.76)	0.53* (CI: 0.26–0.72)
power-mode 3DUS: distal diameter reduction percentage	0.83* (CI: 0.70–0.91)	0.76* (CI: 0.58–0.87)	0.81* (CI: 0.68–0.90)	0.85* (CI: 0.73–0.92)	0.85* (CI: 0.75–0.92)	0.77* (CI: 0.60–0.87)	0.78* (CI: 0.61–0.88)	0.67* (CI: 0.45–0.81)

Reference standard for grading carotid stenosis was 2D colour-coded duplexsonography. Stenotic value in 3D reconstructed ICAS was calculated as distal diameter respectively distal cross-sectional area (CSA) reduction percentage. *ICC* intraclass correlation coefficient, *Ex1* examiner 1, *Ex2* examiner 2, *CI* 95% confidence interval of the intraclass correlation coefficient, *n*.*a*. non available,

* indicates statistical significance i.e. p < 0.05.

Using 2D-CDS as reference standard, positive predictive value for power-mode 3DUS (distal diameter reduction percentage) for detecting a high-grade (≥ 70%) ICAS was 0.81 (Ex1) respectively 0.76 (Ex2) while negative predictive value to exclude a high-grade ICAS was 0.92 (Ex1) respectively 0.91 (Ex2).

## Discussion

In this study we addressed freehand 3DUS for visualisation and quantification of ICAS and found a superiority of power-mode 3DUS in a direct comparison with high-resolution B-mode 3DUS. In detail, the best intra- and inter-rater agreements were achieved for power mode 3DUS and expression of the stenotic value as distal *CSA* reduction percentage, while the best inter-method agreement between power-mode 3DUS and 2D-CDS was found when stenotic value was calculated as distal *diameter* reduction percentage.

Three-dimensional US angiography might ideally complement 2D-CDS for examination of neck vessels which is mainly based on assessment of hemodynamic parameters and is routinely performed in clinical stroke practice. Although 3DUS software algorithms are nowadays implemented in most high-end ultrasound systems they are scarcely used for 3DUS, despite some benefit for examination of carotid vessels which has been demonstrated in previous studies [12, 17]. This is probably due to different technical restrictions associated with the scanning process like the necessity to move the ultrasound transducer with constant velocity and without tilting which is often not achievable in a common ultrasound examination. Moreover, those 3D scans usually do not contain spatial information which excludes offline measurements of the stenosis parameters like diameter or CSA [23]. Ultrasound transducers with a mechanically swept probe have also been used for vascular 3DUS since they can provide such spatial information [11, 13, 16], but their image quality is rather low and only a short vessel segment can be examined.

In our study, freehand 3DUS was achieved by a magnetic field tracking system as an add-on to a conventional ultrasound system. This way, the ultrasound scanning process was identical to a common ultrasound examination of the neck vessels. High spatial and temporal resolution of this system has been demonstrated in before [18]. We found a trend to a higher rate of successful 3D reconstruction of ICAS when using power-mode (93% and 88%) rather than high-resolution B-mode US (81 and 84%). As expected, residual lumen within hypoechogenic ICAS could be visualised better with power mode 3DUS while high-resolution B-mode ultrasound failed to discriminate between lumen and hypoechogenic plaque. On the other hand, heavily calcified plaques with acoustic shadowing resulted in poor image quality within ICAS for both ultrasound modes and likewise restricted 3D vessel reconstruction. These results are in agreement with previous studies where heavily calcified plaques made 3D vessel reconstruction based on power-mode impossible in about 5% of ICAS [10, 12, 14, 15]. To what extent use of ultrasound contrast agents might further increase successful visualisation of ICAS and improve image quality should be addressed in future studies; first results were promising [16].

Inter-rater agreement was clearly better for power-mode 3DUS in comparison to B-mode 3DUS. While the latter showed just a poor to moderate agreement, use of power-mode resulted in a good agreement between both examiners. An even excellent inter-rater agreement was achieved when power-mode 3DUS was used and stenotic value of carotid stenosis was calculated as distal CSA reduction percentage. There are good reasons to express stenotic value of ICAS as distal *CSA* rather than distal *diameter* reduction percentage. First of all, reduction of CSA better represents the hemodynamic impact of—especially an eccentric—stenosis [24]. In addition, measurement of CSA is also independent of the projection, that is, in case of DSA and diameter measurement on the direction of X-rays (Fig 2). Therefore, especially in eccentric carotid stenosis, stenotic values obtained by DSA standard projections might even underestimate the severity of ICAS [25–27]. Even when free rotation of the 3D reconstructed ICAS allows the detection of the smallest stenotic lumen, inter-rater agreement was slightly lower for stenotic values expressed as distal diameter reduction percentage compared to expression as CSA reduction percentage. In contrast, inter-method agreement between power-mode 3DUS based on diameter reduction and 2D-CDS for quantification of ICAS was good for both examiners which is because hemodynamic parameters of the 2D-CDS criteria to grade ICAS were originally adapted to DSA, that is, to distal *diameter* and not *CSA* reduction percentage [8, 9]. This is in line with previous studies which demonstrated the good accuracy of power-mode 3DUS when using diameter reduction in comparison to 2D-CDS and DSA [14, 15].

On the other hand, a moderate and concentric ICAS with a diameter reduction of just 50% would already be of 75% stenotic value when measured as CSA reduction percentage [28]. Hence, it would be difficult to detect a progression over time reliably with conventional ultrasound because 2D section planes would probably vary between examinations [29]. Three-dimensional US enables measurement of CSA perpendicular to the remaining lumen at any point in the vessel’s course, thereby, facilitating follow-up examinations. More importantly, studies that addressed CEA for secondary prevention of symptomatic ICAS used DSA as reference standard and expressed stenotic value as local or distal *diameter* reduction percentage [20, 30, 31]. According to Rothwell and colleagues stenotic value calculated as distal diameter reduction percentage can be transformed to local diameter reduction percentage with good accuracy [32]. However, there is not such a formula for transforming CSA reduction percentage into diameter reduction percentage. Thus, prognostic value of CSA reduction percentage in terms of secondary prevention of symptomatic ICAS is currently unclear. By performing a single power-mode 3DUS examination, benefits of both methods to assess stenotic value could easily be brought together: Carotid stenosis could be described as CSA reduction which would emphasise the hemodynamic aspect and as diameter reduction upon which treatment decision is currently primarily based. Because of good positive respectively negative predictive values of power-mode 3DUS for delineating hemodynamically relevant (≥ 70%) ICAS, it might be used as primary confirmatory imaging modality to support treatment decision. Such an approach of initial 2D-CDS and subsequent power-mode 3DUS would limit more invasive examinations like CTA or MRA to cases where a clear discrepancy is found between both US methods. However, it must be proven if such a strategy is more cost and time effective than the current strategy of 2D-CDS followed by angiography [33, 34].

One limitation of our study is that reference standard for grading ICAS was set to 2D-CDS and not to DSA. In particular, calculation of positive and negative predictive values of 3DUS should refer to DSA which was the imaging modality in the large prospective CEA trials in the 1980’s and 1990’s [20, 30, 31]. However, DSA carries the risk of periprocedural complications [6] and there is a debate, whether DSA can still be regarded as gold standard for grading carotid stenosis [27] since DSA was shown to underestimate stenotic value of ICAS [25, 26]. For carotid stenosis with stenotic values between 70% and 99% sensitivity and specificity of 2D-CDS was demonstrated to be almost equal to MRA and CTA [27]. Moreover, inclusion criteria of ongoing trials like ACST-2 (Asymptomatic Carotid Surgery Trial 2) [35] or ECST-2 (The European Carotid Surgery Trial-2) [36] which examine best treatment of extracranial carotid stenosis allow grading by MRA, CTA and 2D-CDS and do not require DSA at all. Another restriction is that just about one third of ICAS were of ≥ 70% stenotic value, thus, limiting calculation of mainly positive predictive value. Since confirmation of high-grade ICAS is of particular interest in treatment decision, future studies should include more patients with severe carotid stenosis. Finally, because only 9 of the 37 patients had an acute ischaemic stroke or TIA, generalisation of our results to the acute population is limited. Thus, value of 3DUS in the acute phase, where patients are often less compliant and diagnosis might prompt an immediate invasive treatment consequence (e.g. in case of a symptomatic hemodynamically relevant ICAS) must be proven in future studies.

## Conclusion

Our results emphasise superiority of power-mode 3DUS in a direct comparison with B-mode 3DUS for visualisation and quantification of ICAS. Thereby, further studies are warranted which should now compare power-mode 3DUS with the angiographic gold standard imaging modalities for quantification of ICAS, i.e. with CTA or CE-MRA.
